# Identification of Diagnostic and Prognostic microRNAs for Recurrent Vitreous Hemorrhage in Patients with Proliferative Diabetic Retinopathy

**DOI:** 10.3390/jcm8122217

**Published:** 2019-12-15

**Authors:** Parviz Mammadzada, Juliette Bayle, Johann Gudmundsson, Anders Kvanta, Helder André

**Affiliations:** 1 Department of Clinical Neurosciences, Division of Eye and Vision, St. Erik Eye Hospital, Karolinska Institutet, Stockholm 11282, Sweden; parviz.mammadzada@ki.se (P.M.); juliette.bayle@outlook.fr (J.B.); johannrg@landspitali.is (J.G.); anders.kvanta@ki.se (A.K.); 2 Department of Ophthalmology, University of Iceland, Reykjavik 101, Iceland

**Keywords:** proliferative diabetic retinopathy, recurrent vitreous hemorrhage, microRNA-19a, microRNA-20a, microRNA-27a, microRNA-93

## Abstract

MicroRNAs (miRNAs) can provide insight into the pathophysiological states of ocular tissues such as proliferative diabetic retinopathy (PDR). In this study, differences in miRNA expression in vitreous from PDR patients with and without incidence of recurrent vitreous hemorrhage (RVH) after the initial pars-plana vitrectomy (PPV) were analyzed, with the aim of identifying biomarkers for RVH. Fifty-four consented vitreous samples were analyzed from patients undergoing PPV for PDR, of which eighteen samples underwent a second surgery due to RVH. Ten of the sixty-six expressed miRNAs (miRNAs-19a, -20a, -22, -27a, -29a, -93, -126, -128, -130a, and -150) displayed divergences between the PDR vitreous groups and to the control. A significant increase in the miRNA-19a and -27a expression was determined in PDR patients undergoing PPV as compared to the controls. miRNA-20a and -93 were significantly upregulated in primary PPV vitreous samples of patients afflicted with RVH. Moreover, this observed upregulation was not significant between the non-RVH and control group, thus emphasizing the association with RVH incidence. miRNA-19a and -27a were detected as putative vitreous biomarkers for PDR, and elevated levels of miRNA-20a and -93 in vitreous with RVH suggest their biomarker potential for major PDR complications such as recurrent hemorrhage incidence.

## 1. Introduction

Ocular neoangiogenesis is a complex process defined by the formation of new blood vessels from preexisting vasculature [1,2]. This frequently occurs in diabetic retinopathy (DR), a major complication of diabetes mellitus (DM) [3]. Diabetic retinopathy is a microvascular disease in which hyperglycemia plays an important role in the pathogenesis of retinal microvascular damage. Diabetic retinopathy has two stages: non-proliferative diabetic retinopathy (NPDR) and proliferative diabetic retinopathy (PDR). Non-proliferative diabetic retinopathy is the early stage, where apoptosis of pericytes leads to localized microaneurysms. The progressive loss of pericytes, together with endothelial cell damage and dysfunctional capillary basement membrane, contribute to loss of the blood–retina barrier integrity, leading to intraretinal hemorrhages, exudates, and edema of the retinal tissue [3]. Vision acuity is affected when the pathology further involves the macula (i.e., diabetic macular edema) [3,4]. As the disease progresses toward PDR, the autoregulation of capillary blood flow is severely disrupted. Ultimately, this culminates in occlusion of the lumen and/or degeneration of the capillary network, with subsequent ischemia and hypoxia of the retina. At the molecular level, the activation of hypoxia-inducible factor-1 (HIF-1) and subsequent upregulation of proangiogenic factors such as vascular endothelial growth factor (VEGF) are responsible for the impairment of the normal dynamic balance between proangiogenic and antiangiogenic factors, which leads to retinal angiogenesis [3,5,6,7,8]. These neoangiogenic vessels lack in structural integrity, and thus have a tendency to leak and bleed, contributing to preretinal proliferative fibrovascular tissue formation [3,5,7,8]. In advanced cases of PDR, the pathologic retinal vessel proliferation invades the vitreous body, causing sight-threatening vitreous hemorrhage (VH) and/or tractional retinal detachment [3,7,8].

The primary treatment for PDR follows the preventive measures for DM complications with strict glycemic and blood pressure control [7]. In more severe cases, secondary interventions are necessary to prevent vision loss and eventual blindness, which include focal laser photocoagulation and intravitreal angiogenesis inhibitors. Eventually, pars plana vitrectomy (PPV) becomes necessary to clear the vitreous from hemorrhage and to remove the vitreous as a substrate for neovessel ingrowth [9]. To date, the most favorable surgical outcome in PDR seems to result from a successful and timely PPV. Despite successful surgery, the risk of recurrent vitreous hemorrhage (RVH) is high, and approximately 20% of the patients undergo repeated operations within 12 months [10], which illustrates the need for a better understanding of the molecular markers and eventual risk factors present in these patients. RVH after PPV has been associated with elevated vitreous levels of VEGF and angiopoietin 2 [11,12], however, the canonical nature of these factors has made it difficult to employ them as markers for post-surgery complications.

In addition to soluble protein angiogenic factors, the role of microRNAs (miRNAs) has become more pertinent and miRNA expression is related to the development of ocular angiogenesis [13,14]. miRNAs are sequences of small noncoding nucleotides [15,16,17] that are involved in a variety of fundamental cellular processes [18], both intracellularly or secreted via exosomes. miRNAs can play a vital role in pathology, which could define them as promising therapeutic targets [19]. miRNA genes are transcribed and trimmed to form pre-miRNAs with characteristic hairpin structures. Pre-miRNAs are cleaved in the nucleus, exported from the cytoplasm, and processed by Dicer endoribonuclease to yield mature miRNAs. In the cytoplasm, miRNAs post-transcriptionally downregulate gene expression by binding the 3′ untranslated region (3′-UTR) of target mRNAs and marking them for degradation by the RNA-induced silencing complex (RISC) [20]. miRNAs play an important role in the development of the eye, ocular homeostasis, and ocular diseases as their expression levels change in diseases [21,22]. The impact of miRNAs has been demonstrated in several contexts and particularly in antiangiogenic therapies [23]. miRNAs lead themselves as potential biomarkers for the various aspects of DR as they demonstrate considerable stability in biological fluids such as blood and vitreous, facilitating their detection [24,25].

Differential expression of miRNAs in the vitreous of various disease conditions other than PDR has been reported [26,27,28]. Previous studies have analyzed serum and plasma levels of miRNAs in PDR patients with the aim of detecting their relationship to progression of DR or to identify candidate biomarkers [29,30,31,32,33,34], however, some have reported that serum levels of only a few miRNAs correlated to vitreous levels [28,35]. These data imply that studies on ocular biological samples are better candidates to identify prognostic biomarkers, which are still amiss.

In the current study, we analyzed multiple miRNAs in the vitreous of PDR patients after PPV, and illustrated their potential relevance for neoangiogenesis. Furthermore, we evaluated the identified miRNAs as putative biomarkers for PDR, and more importantly, as biomarkers for RVH after PPV.

## 2. Methods

### 2.1. Ethics Approval and Consent to Participate

This study was approved by Stockholm’s regional ethics committee, Sweden (2010-1905-31/1), and written informed consent was obtained before surgery and vitreous sampling. The use of human tissue was compliant with the tenets of the Declaration of Helsinki and General Data Protection Regulation.

### 2.2. Patient Selection

Fifty-four patients of both sexes undergoing PPV were included in the study, with ages ranging from 24 to 83 years (Table 1). The included PDR patients displayed a trend toward type-2 diabetes (T2D) over type-1 (T1D). PDR-associated RVH patients were defined as those undergoing a secondary PPV within a 2-year follow-up. Control vitreous samples were selected from patients undergoing PPV for macular hole or epiretinal membrane removal, conditions known to be unrelated to retinal vascular conditions.

### 2.3. Collection of Vitreous Samples and Grouping

A standard three port 23- or 25-gauge PPV was performed using the Alcon Accurus or Constellation Systems (Alcon Nordic, Stockholm, Sweden). Care was taken not to turn on the infusion before an undiluted vitreous sample of approximately 2 mL was collected and further processed as described below. Peroperative induction of a posterior vitreous detachment, scatter laser photocoagulation, and intravitreal tamponade was applied as per the surgeon’s decision. Undiluted vitreous samples were kept at 4 °C and promptly clarified by centrifugation (14,000 g, 10 min, 4 °C), aliquoted, and stored at −80 °C until analysis.

**Figure 1:**
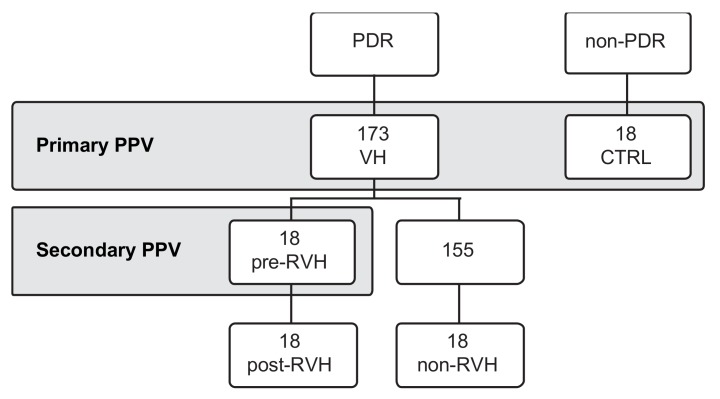
Vitreous samples collection during pars plana vitrectomy (PPV). Vitreous samples were collected from proliferative diabetic retinopathy (PDR) patients or non-PDR controls (CTRL). PDR patients underwent a primary PPV due to vitreous hemorrhage (VH) and were followed for a period of 2-years. A total of 18 patients were reoperated through a second PPV, due to recurrent vitreous hemorrhage (RVH) during the study’s protocol. Vitreous samples from these patients (post-RVH) were paired the primary PPV (pre-RVH). Vitreous from patients that were non-reoperated were categorized as non-RVH.

A total of 191 vitreous samples (173 from PDR patients (VH) and 18 from patients undergoing PPV with known unrelated nonangiogenic ocular conditions (controls)) were collected at the vitreoretinal department of St. Erik Eye Hospital, Stockholm, Sweden. Seventy-two vitreous samples were included in the study for subsequent analyses (Figure 1). With a focus on RVH, the 18 samples resulting from reoperated patients (post-RVH) were paired with their primary PPV vitreous samples (pre-RVH), and compared to 18 randomly selected vitreous samples from non-reoperated patients (non-RVH) and 18 controls. The four groups were matched in sample size and paired to the RVH group as required for analysis (see “Pooled analysis” below). All analyses were performed at the department of molecular and cellular research, St. Erik Eye Hospital.

### 2.4. Detection of miRNA Presence

Fifty μL of each individual vitreous sample from each group were pooled to a total of 1 mL/group (pooled analysis). The miRNeasy Serum/Plasma Kit (Qiagen, Hilden, Germany; Cat No. 217184) was used for the isolation of cell-free total RNA including miRNA from the vitreous, according to the manufacturer’s instructions. The miRNeasy Serum/Plasma Spike-In Control Kit (Qiagen, Cat No. 219610) was used during the extraction protocol. The Kit includes a C. elegans miRNA-39-mimic (Ce), which serves as an isolation efficiency control and internal normalization control for the miRNA profiler assays. The presence of miRNA in each pooled vitreous group was confirmed by running control reactions as described in “Pooled analysis”.

### 2.5. Reverse Transcriptase and Preamplification 

Total RNA containing miRNA was used. Vitreous samples were expected to contain low RNA content, therefore, the miScript PreAMP PCR Kit (Qiagen, Cat No. 331451) was used to prepare cDNA and subsequent preamplification, according to the manufacturer’s instructions. The obtained Ct values allowed for normalization among samples that can control for varying RNA isolation yields and amplification efficiency.

### 2.6. Pooled Analysis

Preamplified cDNA from miRNAs was diluted 20-fold in RNAse-free water and analyzed by real-time PCR for profiling mature miRNA expression. Human miFinder miScript miRNA PCR Array (Qiagen, Cat No. 331221) enabled profiling the expression of the 84 most abundantly expressed human miRNAs (www.miRBase.org), according to the manufacturer’s instructions. The threshold values (Ct) were determined and uploaded into the data analysis software (miScript miRNA PCR Array Data Analysis Excel^®^; http://pcrdataanalysis.sabiosciences.com/mirna), which performs both interpretation of the control assays and quantification using the ∆∆Ct method. The expressed miRNAs were compared between the vitreous sample groups and fold differences were obtained for each miRNA. As recommended by the manufacturer, miRNAs with Ct values above 35 were excluded; relative expressions with a threshold above 4-fold were considered different and selected for further analysis.

### 2.7. Single-Patient Analysis

miRNA isolation was repeated on each of the 72 patients’ vitreous samples separately (170 μL/sample). The single-patient miRNAs were reverse transcribed and preamplified into cDNAs as described above including preamplified cDNA confirmation. miRNAs (miRNA-19a; -20a; -22; -27a; -29a; -93; -126; -128; -130a; -150) and the Ce miRNA spike-in as the internal control were profiled by a customized miScript Primer Assay (Qiagen). Data was analyzed by the ΔΔCt method as described in “Pooled analysis”.

### 2.8. Statistical Analysis

Pooled analysis was performed descriptively as a form of substantial expression comparison between the study groups by the ΔΔCt method. Subsequently, analysis of the differentially expressed miRNAs from the pooled analysis was assayed on a single-patient basis (*N* = 18 per group) and represented as relative expression (2^−ΔCt^). Data were presented as box-plots with Tukey correction, and statistically analyzed by one-way ANOVA with Fisher’s multiple comparison posttest (*p* < 0.05 was considered significant).

## 3. Results

### 3.1. Angiogenic miRNAs Profiling Illustrated Divergences within Pooled Proliferative Diabetic Retinopathy (PDR) Vitreous

**Figure 2:**
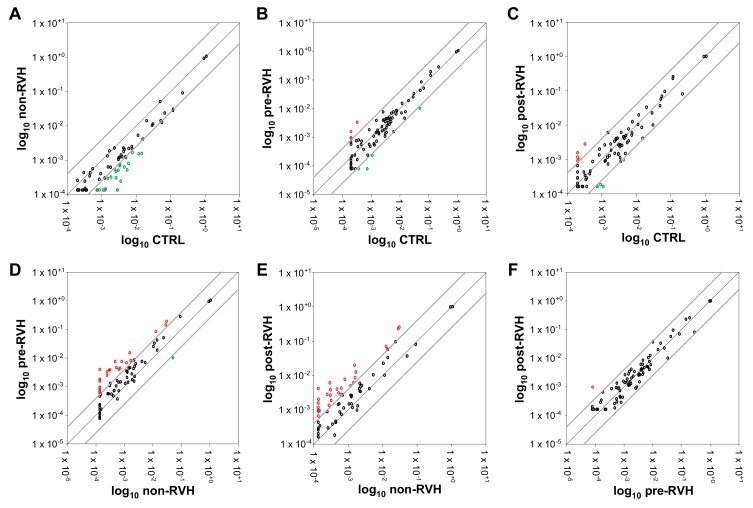
Relative regulation of angiogenic miRNA expression in PDR and control vitreous. Pooled vitreous samples from nonangiogenic controls (CTRL), and non-RVH, pre-RVH, and post-RVH PDR groups were analyzed by miRNA PCR array using the ΔΔCt method. A threshold of 4-fold differential regulation was applied and data presented as correlation scatter plots for non-RVH versus CTRL (**A**), pre-RVH vs. CTRL (**B**), post-RVH versus CTRL (**C**), pre-RVH versus non-RVH (**D**), post-RVH versus non-RVH (**E**), and post-RVH versus pre-RVH (**F**). Refer to Table S1 for specific ΔΔCt values.

Pooled samples from the four vitreous sample groups were analyzed by the miRNA PCR array for the purpose of identifying up- and downregulated miRNA expression among the vitreous groups. A 4-fold relative regulation cut-off was applied to the comparisons (Figure 2) to facilitate the putative utility of the identified miRNAs as clinically available biomarkers. The expression of 66 out of the 84 profiled miRNAs was detected in all groups (Table S1). Only downregulated miRNAs were identified by the comparison of non-RVH PDR vitreous to the control (Figure 2A), while pre-RVH or post-RVH PDR samples identified a small cluster of upregulated miRNAs when compared to the control vitreous (Figure 2B,C).

To assess RVH-associated biomarkers, PDR samples from patients affected by RVH were compared to non-RVH samples. A noticeable group of miRNAs was upregulated both in pre- and post-RVH PDR vitreous in comparison to non-RVH vitreous (Figure 2D,E). To focus on miRNAs relevant for RVH, PDR vitreous from such patients were analyzed comparatively (Figure 2F), and one miRNA indicated an upregulation in post-RVH compared to pre-RVH pooled samples.

**Figure 3:**
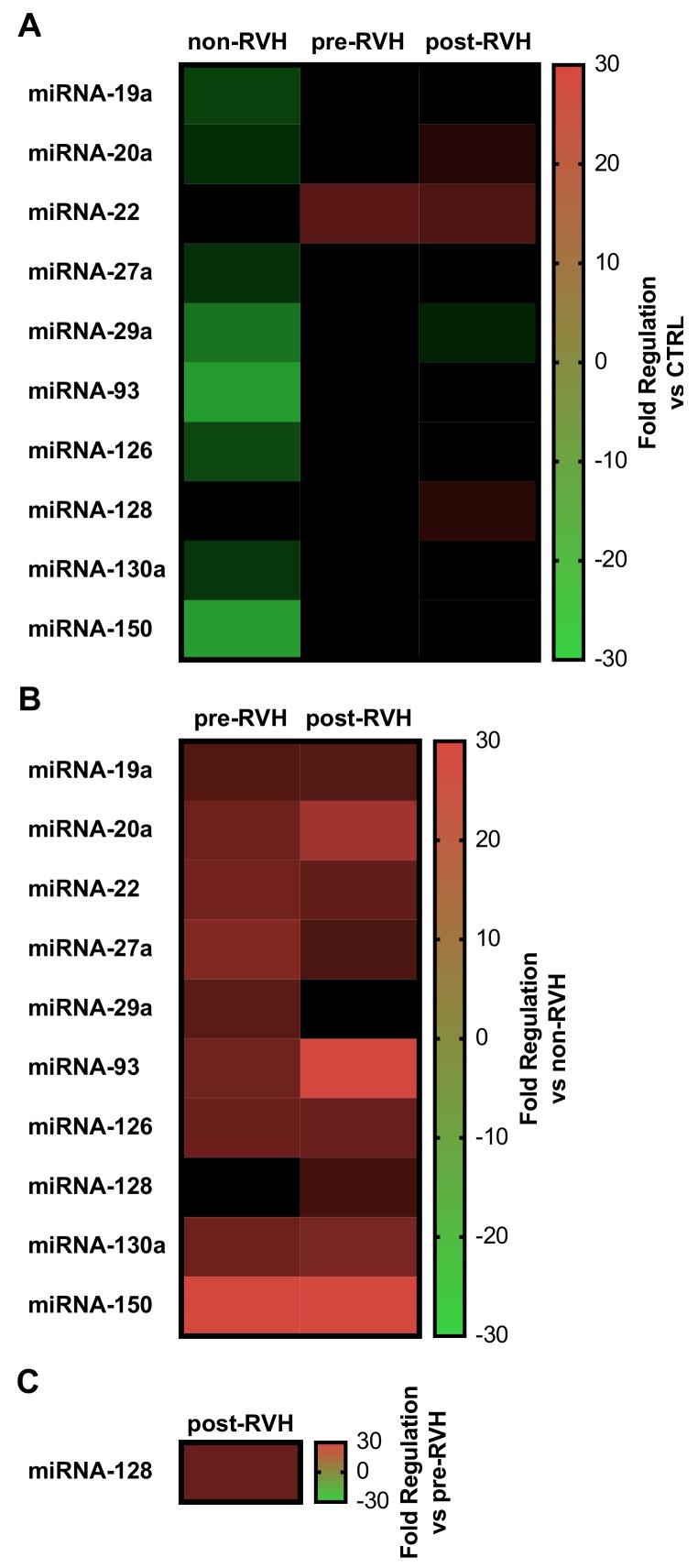
Heatmaps of relevant angiogenic miRNA expression in PDR vitreous. Relative fold regulation from pooled vitreous from the controls (CTRL), non-RVH, pre-RVH, and post-RVH were analyzed by the ΔΔCt method and displayed as comparative matrixes versus CTRL (**A**), versus non-RVH (**B**), or versus pre-RVH (**C**). For comparison, the scale was normalized and represented as positive values for upregulated miRNAs (red), while negative for downregulations (green).

Taken together, these results demonstrated a cluster of miRNAs that differed considerably among the samples analyzed. Using heatmaps of correlated ΔΔCt values (Figure 3), miRNAs of interest could be compared among groups.

Pooled vitreous from non-RVH PDR patients only displayed decreased expression of angiogenic miRNAs, with miRNA-93 and miRNA-150 presenting the highest downregulation (20.1- and 21.2-fold, respectively; Figure 3A). Nevertheless, vitreous from patients undergoing PPV due to VH (pre-RVH) demonstrated increased expression of miRNAs when compared to nonangiogenic controls, specifically miRNA-22 (10-fold), while vitreous from PDR patients afflicted with RVH—post-RVH—displayed upregulation of miRNAs-20a, -22, and -128 (4.0-, 8.6-, and 4.4-fold respectively; Figure 3A).

With a focus on identifying miRNAs that could be correlated to the risk of RVH, pooled vitreous from pre- or post-RVH group were compared to the non-RVH group (Figure 3B). miRNA-19a, -20a, -22, -27a, -29a, -93, -126, -128, -130a, and -150 indicated a possible correlation to the occurrence of RVH after initial PDR-associated VH. miRNA-150 showed the highest upregulation in both pre-RVH (29.3-fold) and post-RVH (30.6-fold) PDR vitreous, with miRNA-19a, -20a, -22, -27a, -93, -126, and -130a also considerably upregulated (respectively per pre- and post-RVH; 9.5- and 9.6-fold; 13.1- and 20.7-fold; 13.5- and 11.5-fold; 16.0- and 8.6-fold; 13.2- and 30.4-fold; 12.6- and 12.4-fold; and 13.1- and 14.6-fold) versus non-RVH group. Of interest, miRNA-29a was upregulated (10.3-fold) in pre-RVH, but not in post-RVH PDR vitreous when compared to non-RVH vitreous. Finally, to further illustrate molecular events relevant for RVH, only miRNA-128 showed increased expression (12.1-fold) in samples from PDR patients upon post-RVH when compared to the pre-RVH group (Figure 3C).

In sum, ten of the sixty-six expressed miRNAs (miRNA-19a, -20a, -22, -27a, -29a, -93, -126, -128, -130a, and -150) displayed potential divergences between the PDR vitreous groups and to the control, and were further analyzed by relative expression on a single-patient basis.

### 3.2. miRNA-19a and miRNA-27a Are Increased in PDR Patients, and Are Decreased in PDR Patients Undergoing Secondary pars plana vitrectomy (PPV)

**Figure 4:**
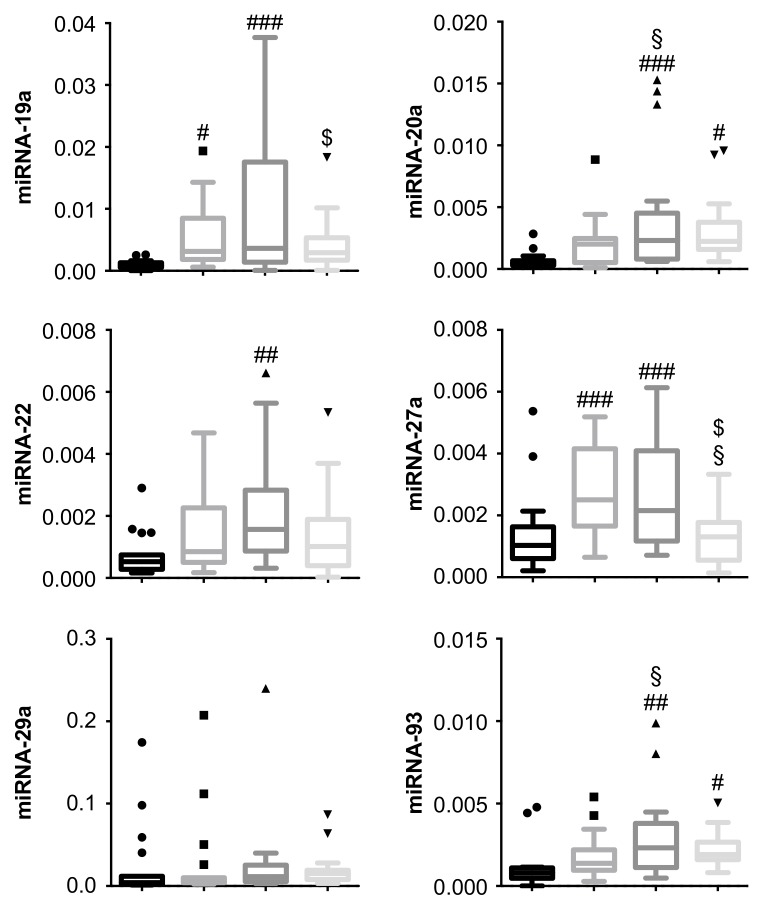

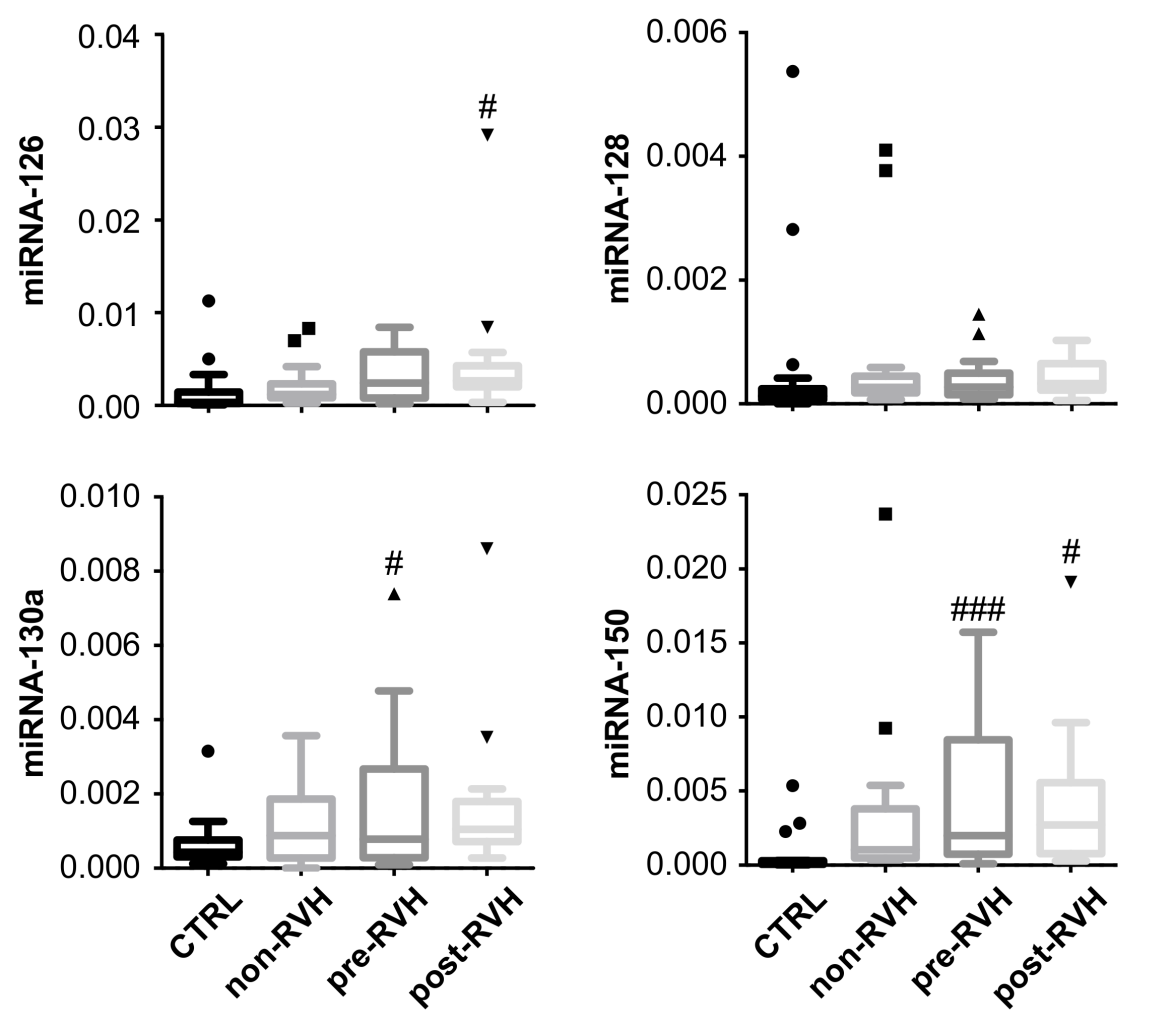
Expression patterns of angiogenic miRNA in vitreous from the PDR and control patients. miRNA expression levels were evaluated by quantitative PCR and analyzed as relative expression (2^−ΔCt^) using Ce miRNA spike-in as the internal standard. Data represented as box-plots with Tukey correction (*N* = 18 per group). # *p* < 0.05, ## *p* < 0.01, ### *p* < 0.001 versus control (CTRL); § *p* < 0.05 versus non-RVH; $ *p* < 0.05 versus pre-RVH (one-way ANOVA with Fisher’s post hoc tests).

The identified 10 miRNAs were isolated and analyzed by qPCR for each of the 72 patients’ vitreous samples separately, according to their respective grouping. miRNA expression was determined based on each patient’s ΔCt values to the internal standard (Figure 4).

Particularly, miRNA-19a and miRNA-27a were strongly upregulated between patients with PDR when compared to nonangiogenic controls (*p* < 0.001; with the exception of miRNA-19a non-RVH versus the control, *p* < 0.05). miRNA-20a and -150 also identified in the pooled analysis were significantly upregulated versus the control patients (*p* < 0.001 for pre-RVH and *p* < 0.05 for post-RVH patients), but upregulation was not significant (*p* = 0.1 in both) in the non-RVH patients versus the control, lessening their potential as vitreous biomarkers for PDR.

Within the 2-year follow-up period, a subgroup of PDR patients suffered a RVH and were reoperated through a secondary PPV. miRNA-19a and miRNA-27a were significantly reduced in these patients (i.e., post-RVH group), which could suggest a putative role for these miRNAs in RVH.

### 3.3. miRNA-20a and miRNA-93 Are Upregulated in PDR Patients with Recurrent Vitreous Hemorrhage (RVH) in Single-Patient Analysis

Vitreous samples of patients undergoing primary PPV were subdivided between patients that were non-reoperated—non-RVH—or were reoperated due to recurrent hemorrhage—pre- and post-RVH—during the 2-year follow-up period (Figure 4). In fact, both miRNA-20a and miRNA-93 were significantly increased (*p* < 0.05) in vitreous from post-RVH patients when compared to non-RVH patients. The levels did not differ significantly (*p* = 0.11) between the non-RVH and control group, thus emphasizing the association of miRNAs-20a and -93 with RVH.

Expression differences of five miRNAs (miRNA-22a, -29a, -126, -128, -130a) could not be confirmed among PDR vitreous samples in single-patient analysis compared to pooled analysis. No significant expression differences were observed for miRNA-29a and -128 between the groups; despite the observed upregulation in the pooled samples (Figure 2C), this could not be confirmed statistically in single-patient analyses. miRNA-126 were significantly upregulated in the post-RVH group vs. control, and miRNAs-22a and -130a were upregulated in the pre-RVH group compared to the control, but no significant expression differences could be confirmed statistically between the PDR vitreous groups (pre-RVH versus post-RVH), diminishing their relevance as biomarkers in PDR-associated RVH.

## 4. Discussion

The study presented here is the first to analyze miRNAs in human vitreous PDR samples with regard to PPV and RVH. Several risk factors including remaining or recurrent proliferative neovascular membranes and fibrovascular ingrowth at the sclerotomy sites are associated with RVH in this population [36]. Nonetheless, the molecular mechanisms of RVH are yet to be elucidated. Expression of 84 miRNAs in vitreous samples of 72 patients, subdivided in four groups—nonangiogenic controls; non-RVH; and pre- and post-RVH—was determined using pooled-sample analysis. Of the analyzed miRNAs, 10 (miRNA-19a, -20a, -22, -27, -29a, -93, -126, -128, 130a, and -150) were identified that may play a role in angiogenesis with a focus on PDR-associated RVH. Upon confirmation by single-patient analysis, miRNA-19a and -27a were shown to be increased in the vitreous of patients with PDR when compared to the nonangiogenic controls. In addition, miRNA-20a and -93 correlated with RVH incidence as they were significantly elevated in patients undergoing primary PPV and later afflicted by RVH.

Both miRNA-19a and -27a were significantly increased when compared with the nonangiogenic controls. miRNA-19a is highly expressed in endothelial cells, being strongly upregulated by ischemia, and its elevated levels are associated with DR in rats [37,38]. miRNA-27a has been shown to have protective roles in angiogenic and inflammatory conditions observed in PDR [39,40]. Combined, our data suggest that vitreous miRNA-19a and miRNA-27a may serve a role as diagnostic biomarkers in PDR. Furthermore, the decreased expression of these miRNAs in the post-RVH group observed in the present study suggests additional roles for both miRNA-19a and 27a in RVH through the scarcity of intrinsic protective factors.

The association of miRNA-20a and -93 in PDR have previously been proposed [41]. Chen et al. analyzed the aqueous humor of PDR patients in comparison to nonangiogenic controls, finding 484 miRNAs differentially expressed with only eight miRNAs significantly different (miRNA-16-2, -30c, -93, -99b, -140, -150, -1827, novel_mir4, novel_mir340). In our study, we found that miRNA-93 and -150 were among the miRNAs displaying significantly increased expression, albeit previously reported to be downregulated in the aqueous humor of PDR patients. This discrepancy could be due to anatomical differences between the aqueous and vitreous humors. It is reasonable to imagine that the pathophysiological milieu is different between the anterior and posterior chambers of the eye, particularly regarding proximity to the retinal tissue.

To date, few studies have analyzed miRNAs in vitreous from PDR patients compared to nonangiogenic controls [35,42,43]. Hirota et al. analyzed 168 miRNAs in four PDR vitreous samples in comparison to four macular hole (MH) controls, and in correlation to serum levels [35]. Coherently, significant upregulation of miRNA-93 in PDR vitreous was demonstrated in the present study. Usui-Ouchi et al. analyzed 377 miRNAs in three vitreous of patients with proliferative vitreoretinal disease including PDR, compared to MH [42]. The authors showed that both miRNA-20a and -93 displayed a tendency, although not significantly, to be elevated in PDR, which corroborated with the results presented here, where significant increased expression was demonstrated. Weak statistics in the previous authors’ data were probably due to the smaller sample size compared to the data presented herein (*N* = 3 vs. *N* = 18). Overall, the present study provides deepened insight into the association of miRNA-20a and -93 in PDR through the analysis of their expression in a larger sample size (*N* = 18). In addition, and for the first time, by further sub-grouping the PDR vitreous according to rebleeding incidence, the present study hints at the potential role of miRNA-20a and -93 as prognostic biomarkers for RVH associated with PDR.

Both miRNA-20a and -93 are miRNA-17 family members (includes miRNAs-20a/b, -93, and -106a/b), which are known to regulate angiogenesis, specifically the HIF/VEGF pathway [44,45], and thus candidates as biomarkers for PDR-associated RVH. miRNA-20a has been suggested to act as an antiangiogenic regulator and is induced under hypoxic conditions, targeting angiogenic factors produced by endothelial cells including VEGF and HIF-1α [45,46,47]. Crosstalk between HIF-1α and VEGF through interactions with their common miRNAs has been demonstrated in a mouse model of DR [45]. miRNA-20a overexpression significantly reduced the expression of both VEGF and HIF-1α protein levels in a retinal pigment epithelium cell-line (ARPE-19) exposed to hyperglycemia and hypoxia/reoxygenation [45], and elevated levels of miRNA-20a in serum have been correlated with the progression of DR in patients [48]. Overall, the experiments suggest a role for miRNA-20a in the pathogenesis of PDR through miRNA crosstalk of HIF-1α and VEGF, and moreover, as a potential biomarker for PDR complications, specifically RVH incidence, as demonstrated for the first time by the present data.

Characterization of miRNA-93 has suggested its ambiguous role in angiogenesis, since it potentially targets a great number of genes, and can both induce and inhibit angiogenesis. However, a notable number of studies indicate that miRNA-93 downregulates VEGF expression through binding to the 3′-UTR of the *Vegfa* gene (reviewed in [49]). Nonetheless, elevated expression of miRNA-93 upon analysis from vitreous and serum appeared to be associated with PDR progression and complications [28,29].

The imbalance among angiogenic molecules such as VEGF and others, causing the hemorrhagic tendency observed in RVH-afflicted PDR patients, could trigger antiangiogenic signals. Thus, we postulate that elevated levels of miRNA-20a and -93 occur due to their mechanism of action as negative regulators and compensatory antiangiogenic effects.

Interestingly, the present data could not corroborate significant differential expression for certain miRNAs previously reported as associated with PDR [25,50,51]. miRNA-29a has been shown to be a negative regulator of DR and its decrease in expression levels is associated with exacerbation of PDR [25,51]. The absence of compensatory upregulation of miRNA-29a in PDR vitreous could be linked to the progression of PDR, thus leading to vitreous hemorrhage. miRNA-128 regulates multiple pathways within the cell including pathways involved in cancer or neuronal maturation. It has been shown to have tumor suppression activity, and has received particular interest in neuroscience research as it is highly expressed in the cortex and cerebellum during development [50]. Detection of miRNA-128 expression in human vitreous is reported here for the first time, however, its relevance as a biomarker or molecular effector of ocular disease including PDR needs further study.

miRNA-126 has been extensively studied in DR and is considered to be endothelial cell-specific, inhibiting different cascades of angiogenesis including cell migration, reorganization of the cytoskeleton, capillary network stability, and cell survival in vitro. miRNA-126 overexpression inhibits high glucose-induced migration and tube formation of rhesus macaque choroidal and retinal endothelial cells by blocking *Vegfa*, and intravitreal injection of miRNA-126 reduced the levels of VEGF, IGF-2, and HIF-1α [38,52,53].

In the present study, elevation of miRNA-126 was not detected in the vitreous of non-RVH or pre-RVH compared to the control, while it was elevated in post-RVH. These data led us to hypothesize that the delayed onset of protective miRNA-126 could contribute to complications of PDR such as RVH.

We demonstrated significantly elevated levels of miRNA-22, -130a and -150 in pre-RVH and/or post-RVH groups compared to the control, with nonsignificant upregulation compared to non-RVH. These miRNAs have been shown to have negative regulatory effects on angiogenesis and protective roles against DR [54,55,56,57]. Elevated levels in only PDR vitreous that recurrently hemorrhage (detected during both first and second PPV) may suggest insufficient compensatory mechanisms, thus putatively causing RVH.

It is noteworthy that previous studies suggested the vitreous to be a more reliable substrate for miRNA analysis than serum/plasma, since the serum levels of only a few miRNAs could be correlated to vitreous levels [28,35]. In a study by Hirota et al., differential expression pattern for miRNA-93 in serum could not be confirmed, despite significant upregulation in the vitreous [35]. Taken together, post-operative vitreous samples appear superior for this purpose. Furthermore, in the current study, the biological functions of the detected miRNAs in relation to PDR pathogenesis were not addressed, since most of the identified miRNAs including miRNA-93a and miRNA-20a have been well characterized previously in DR, as discussed above.

The present study suggests the biomarker potential of miRNA-20a and miRNA-93. Furthermore, the data here demonstrate that significant upregulation of miRNA-20a and miRNA-93 may be related to RVH incidence, as differences between the non-RVH vs. control group in the single-patient analysis were non-significant. Consequently, these findings further emphasize that elevated levels of miRNA-20a and -93 are not associated with leakage from the circulation during vitreous hemorrhagic events, but rather that their presence in the vitreous is either active secretion from retinal cells including pathologic fibrovascular/angiogenic tissues, or leakage from necrotic/apoptotic cells. Further studies are needed to confirm the association with RVH either in vitro and/or in vivo, and to analyze absolute concentrations of miRNA-20a and -93 from vitreous samples, in order to establish reference values for future screening analysis.

## 5. Conclusions

Upon profiling 84 different human miRNAs from a large sample of 72 vitreous, the present study detected an association of several miRNAs with PDR pathology. miRNA-19a and miRNA-27a combined are putative diagnostic biomarkers for PDR. miRNA-20a and miRNA-93 are significantly elevated in vitreous from patients afflicted with RVH, suggesting a prognostic biomarker potential for sight-threatening PDR complications such as RVH.

## Figures and Tables

**Table 1:** Demography of patients for whom vitreous analysis was performed.

Patient Variables	Control	Non-Reoperated ^a^	Reoperated ^b^
*N*	18	18	18
Age (mean, range) ^c^	69 (29–80)	58 (39–83)	53 (24–81)
Sex (M/F) ^d^	12/6	12/6	8/10
Surgery (MH/ERM) ^e^	4/14		
DM type (T1D/T2D) ^f^		7/11	8/10
DM duration (mean, range) ^g^		24 (5–41)	21 (1–44)
Rubeosis iridis (*N*) ^h^		2	3
Neovascular glaucoma (*N*) ^i^		1	0
Preoperative laser (*N*) ^j^		16	16
Peroperative laser (*N*) ^k^		17	15
PVD (*N*) ^l^		6	3
Tamponade (n/a/s/c) ^m^		1/10/4/3	3/10/1/4
2nd PPV (mean, range) ^n^			13 (2–48)

^a^ PDR cases without recurrent postoperative vitreous hemorrhage; ^b^ PDR cases reoperated due to recurrent postoperative vitreous hemorrhage; ^c^ Age is given in years; ^d^ Male/Female; ^e^ Macular hole (MH); epiretinal membrane (ERM); ^f^ type-1 diabetes (T1D); type-2 diabetes (T2D); ^g^ Duration is given in years; ^h^ Preoperative rubeosis iridis present; ^i^ Preoperative neovascular glaucoma present; ^j^ Preoperative scatter laser was present; ^k^ Preoperative scatter laser present; ^l^ Complete preoperative posterior vitreous detachment (PVD) present; ^m^ Surgical tamponade used (none/air/SF6/C3F8); ^n^ Duration until secondary PPV is given in weeks.

## Supplementary Materials

The following are available online at https://www.mdpi.com/2077-0383/8/12/2217/s1, Table S1: Relative regulation of angiogenic miRNA between PDR and the control vitreous, over a 4-fold cut-off.
